# A highly selective and sensitive near-infrared fluorescent probe for imaging of hydrogen sulphide in living cells and mice

**DOI:** 10.1038/srep18868

**Published:** 2016-01-08

**Authors:** Ling Zhang, Xi Emily Zheng, Fang Zou, Yanguo Shang, Wenqi Meng, En Lai, Zhichen Xu, Yi Liu, Jing Zhao

**Affiliations:** 1 School of Chemistry and Chemical Engineering, Nanjing University, Nanjing, 210093, China; 2 Jiangsu Key Laboratory of New Drug Research and Clinical Pharmacy, School of Pharmacy, Xuzhou Medical College, Xuzhou, 221002, China; 3 Guangdong Key Lab of Nano-Micro Material Research, School of Chemical Biology and Biotechnology, Peking University Shenzhen Graduate School, Shenzhen, 518055, China; 4 Department of Gastroenterology, Nanjing Drum Tower Hospital, The Affiliated Hospital of Nanjing University Medical School, Nanjing, 210029, China

## Abstract

Hydrogen sulphide (H_2_S), the third endogenous gaseous signalling molecule, has attracted attention in biochemical research. The selective detection of H_2_S in living systems is essential for studying its functions. Fluorescence detection methods have become useful tools to explore the physiological roles of H_2_S because of their real-time and non-destructive characteristics. Herein we report a near-infrared fluorescent probe, NIR-HS, capable of tracking H_2_S in living organisms. With high sensitivity, good selectivity and low cytotoxicity, NIR-HS was able to recognize both the exogenous and endogenous H_2_S in living cells. More importantly, it realized the visualization of endogenous H_2_S generated in cells overexpressing cystathionine β-synthase (CBS), one of the enzymes responsible for producing endogenous H_2_S. The probe was also successfully applied to detect both the exogenous and endogenous H_2_S in living mice. The superior sensing properties of the probe render it a valuable research tool in the H_2_S-related medical research.

Hydrogen sulphide (H_2_S) has emerged as the third endogenous gaseous signalling molecule in living organisms, along with nitric oxide (NO) and carbon monoxide (CO)^1^,^2^. It is mainly produced by enzymes such as cystathionine β-synthetase (CBS), cystathionine γ-lyase (CSE) and cysteine aminotransferase (CAT)/3-mercaptopyruvate sulphurtransferase (3-MST)^3^. Physiological levels of H_2_S have diverse biological functions, including neurotransmission^4^, vasodilation^5^, apoptosis^6^, inflammation^7^, ischemia/reperfusion-induced injury^8^ and insulin secretion^9^. In addition, abnormal concentrations of H_2_S appear to be involved in many diseases^10^,^11^, such as Alzheimer’s disease and diabetes mellitus.

With increasing interest in understanding the chemical and biological properties of H_2_S, sensitive and selective detection techniques for monitoring endogenous H_2_S are urgently desirable, since the complex manifestations of H_2_S in both physiological and pathological states, as well as its underlying molecular events are still not fully understood. The current approaches for H_2_S detection, such as the methylene blue method, the monobromobimane (MBB) method, gas chromatography (GC), and the sulphide ion selective electrodes (ISE) method are not suitable for *in situ* analysis^12^. Fluorescence-based assays, however, could offer convenience, high sensitivity, nondestructiveness, as well as real-time imaging^13^.

Recently, various sensing strategies have been focused on the design of H_2_S-reactive probes, including nucleophilic addition^14^,^15^,^16^,^17^,^18^,^19^,^20^, copper sulphide precipitation^21^,^22^,^23^, H_2_S-mediated reduction^24^,^25^,^26^,^27^,^28^,^29^, and the thiolysis of dinitrophenyl ether by H_2_S^30^,^31^, *etc*^32^. However, most of these fluorescent probes are based on fluorophores with peaks in the ultraviolet-visible (UV/V is) region, which renders them difficult to be employed for imaging H_2_S in living animals due to high absorption and autofluorescence of biomolecules^33^. By contrast, probes with absorption and emission in the near-infrared (NIR) region are more desirable for *in vivo* imaging because of minimal photo damage, deep tissue penetration, and minimum interference from background autofluorescence^34^. Recently, several research groups have reported excellent NIR fluorescent probes for H_2_S^35^,^36^,^37^,^38^,^39^,^40^,^41^,^42^,^43^. Lin *et al.* prepared a NIR fluorescent probe by utilizing thiolysis of dinitrophenyl ether^38^. Tang *et al.* constructed a NIR ratiometric fluorescent probe for rapid imaging of endogenous H_2_S *via* nucleophilic addition with an impressive detection limit of 5.0–10.0 nM^40^. Guo *et al.* introduced a NIR fluorescent probe based on selective complexation ability with copper^41^. Despite these progresses, we are interested in developing practical NIR probes with a combination of desirable characteristics, especially low detection limits and the ability to detect endogenous H_2_S in living animals. Fluorescent probes with a lower detection limit, especially the nanomole range, or even lower, are needed owing to the low levels of endogenous H_2_S in cells/plasma/tissues. Moreover, the probes for detection of endogenous H_2_S *in vivo* are still sparse^44^,^45^. Thus, NIR probes with higher sensitivity and favourable properties to monitor endogenous H_2_S *in vivo* are in high demand.

Herein, we prepared NIR-HS as a NIR fluorescent probe for H_2_S detection. The key features of NIR-HS include good selectivity, high sensitivity and suitability for recognizing endogenous H_2_S in living cells and mice.

## Results and Discussion

### Synthesis and sensing mechanism of NIR-HS

**Figure 1:**
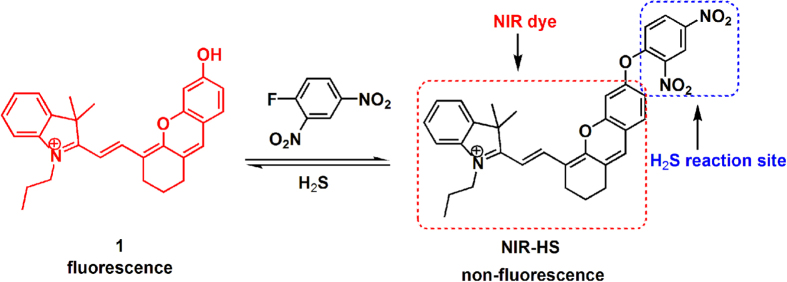
Design and synthesis of NIR fluorescent turn-on probe NIR-HS.

In the design of a NIR probe for H_2_S, the hemicyanine skeleton (a NIR dye) was selected as a fluorophore in the light of its NIR emission and high stability^46^. It is known that the thiolysis of the dinitrophenyl ether reaction can be chemoselective for H_2_S over biothiols^38^. Thus, probe NIR-HS was constructed by connecting a dinitrophenyl group to hemicyanine (a NIR dye) via an ether-linkage (Fig. 1). The fluorescence of NIR-HS was quenched due to alkylation on the hydroxyl group^46^. We speculated that the reaction of sulphide with NIR-HS would cleave an ether group, and release the free fluorophore, thereby achieving fluorescence detection of sulphide. On the basis of this design, the structure of NIR-HS and the proposed sensing mechanism are illustrated in Fig. 1. The probe was readily synthesised in two steps. Treatment of IR-780 with resorcin in the presence of K_2_CO_3_ afforded compound 1, which was then condensed with 1-fluoro-2,4-dinitrobenzene to generate target compound NIR-HS (please refer to the Supplementary Information for details). Finally, target probe, NIR-HS, was characterized by NMR spectroscopy and mass spectrometry (please refer to the Supplementary Information for details).

The reaction of NIR-HS with sulphide produced a red fluorescent product, which was identical to the absorption and emission of the authentic compound 1 (Supplementary Fig. S1). Moreover, the thiolysis product was characterised by HRMS and ^1^H NMR spectra (please refer to the Supplementary Information for details), demonstrating that the reaction of NIR-HS with sulphide proceeded as designed in Fig. 1.

### Fluorescent properties of NIR-HS

**Figure 2:**
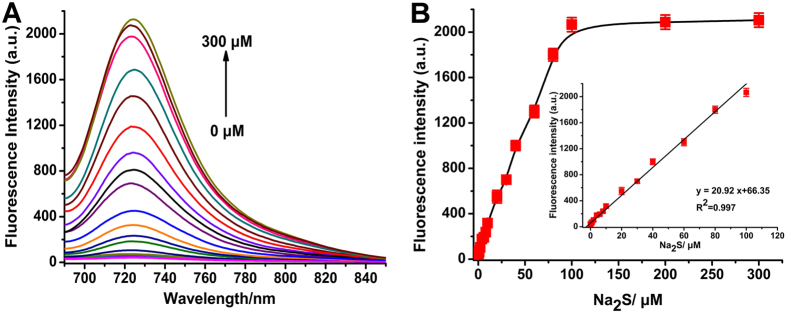
Fluorescence response of NIR-HS to sulphide. (**A**) Fluorescence spectra of NIR-HS (10 μM) with Na_**2**_S (0, 0.05, 0.1, 0.2, 0.4, 0.6, 0.8, 1, 2, 4, 6, 8, 10, 20, 30, 40, 60, 80, 100, 200 and 300 μM) in PBS buffer (20 mM, pH = 7.4, 5% CH_3_CN) at 37 °C for 20 min. (**B**) The fluorescence intensity changes of NIR-HS (10 μM) at 723 nm after incubation with different concentrations of Na_2_S (0–300 μM). Insert: The linear relationship between the fluorescence intensity and the concentrations of Na_2_S (0 to 100 μM) in PBS buffer. Data are presented as the mean ± SD (*n* = 3).

The fluorescent properties of NIR-HS (10 μM) in the absence and presence of Na_2_S were determined. The free probe was almost nonfluorescent (Fig. 2A). However, treatment of Na_2_S (100 μM) led to a large fluorescence enhancement at 723 m (50 fold, *Φ* = 0.13). Figure 2B depicted elevated fluorescence intensities with increasing amounts of Na_2_S (0–300 μM) until a plateau reached at 100 μM Na_2_S. An excellent linear correlation between the observed fluorescence intensities and various concentrations of Na_2_S (0–100 μM) was observed in PBS buffer (Fig. 2B inset). The *in vitro* detection limit for sulphide was 38 nM, which was lower than most of the reported NIR H_2_S probes. Therefore, NIR-HS is highly sensitive to low-nanomolar levels of sulphide, which facilitate the quantitative detection of endogenous/intracellular H_2_S in complex biological systems.

The fluorescence intensity in reaction of NIR-HS with Na_2_S reached the maximum value within approximately 20 min (Supplementary Fig. S2). The effects of pH on the detection of sulphide were then evaluated (Supplementary Fig. S3). In the pH range from 5.8 to 6.0, the emission intensities were quite low and did not change significantly. From pH 6.2 to 6.8, the fluorecence intensities were gradually increased, and the maximal fluorecence intensities were observed from pH 7.0 to 9.0. The emission profile of fluorophore (compound **1**) (Supplementary Fig. S4) are consistent with the results of treating the probe with Na_2_S in different pH PBS buffer, indicating that the observed pH profile is due to the fluorophore itself. Taken together, NIR-HS is suitable for the detection of sulphide between pH 7.0 and 9.0.

**Figure 3:**
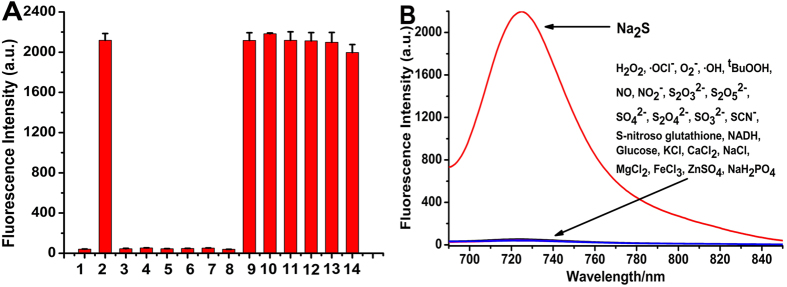
The selectivity of NIR-HS for sulphide. (**A**) Fluorescence responses of NIR-HS (10 μM) towards Na_2_S (100 μM) and various biothiols after 20 min of incubation. 1. Na_2_S (0 μM); 2. Na_2_S(100 μM); 3. Hcy (100 μM); 4. GSH (1 mM); 5. Cys (100 μM); 6. Cys (1 mM); 7. GSH (10 mM); 8. Hcy (1 mM); 9. Na_2_S (100 μM) + Hcy (100 μM); 10. Na_2_S (100 μM) + Hcy (1 mM); 11. Na_2_S (100 μM) + Cys (100 μM); 12. Na_2_S (100 μM) + Cys (1 mM); 13. Na_2_S (100 μM) + GSH (1 mM); and 14. Na_2_S (100 μM) + GSH (10 mM). (**B**) Fluorescence spectra of NIR-HS (10 μM) towards Na_2_S (100 μM), reactive oxygen species, reactive nitrogen species, sulphur-containing inorganic ions, S-nitroso glutathione (SNG), reducing agents and inorganic salts (1 mM) after 20 min of incubation. Data are presented as the mean ± SD (*n* = 3).

**Selectivity to sulphide of NIR-HS.** To investigate the selectivity of NIR-HS towards sulphide, NIR-HS was treated with various species. NIR-HS displayed high selectivity for H_2_S over physiological concentrations of biological thiols (Fig. 3A and Supplementary Fig. S5), including glutathione (10 mM GSH), cysteine (1 mM Cys) and homocysteine (1 mM Hcy). The good selectivity of NIR-HS attribute to the stronger nucleophilic properties of HS^−^, smaller size and a lower pKa at neutral pH compared to other thiols. The remaining non-thiol amino acids (Ala, Glu, Trp, Met, Tyr, Leu, Val, Ser, Pro, Arg, Gly, Phe, His, Gln, Asn, Ile and Thr), reactive oxygen species (H_2_O_2_, ·OCl^−^, O_2_^−^, ·OH and ^t^BuOOH), reactive nitrogen species (NO_2_^−^ and NO), sulphur-containing inorganic ions (S_2_O_3_^2−^, S_2_O_5_^2−^, SO_4_^2−^, S_2_O_4_^2−^, SO_3_^2−^ and SCN^−^), S-nitroso glutathione (SNG), reducing agents (NADH and glucose) and inorganic salts (KCl, CaCl_2_, NaCl, MgCl_2_, FeCl_3_, ZnSO_4_ and NaH_2_PO_4_) induced negligible responses (Fig. 3B and Supplementary Fig. S6). Additionally, competitive experiments also revealed hardly any interference to sulphide detection in the coexistence of Na_2_S and various species (Supplementary Fig. S6 and Fig. S7). The fluorescence intensity decreased in the presence of H_2_O_2_ and ZnSO_4_ due to the oxidation of H_2_S by H_2_O_2_ and sulphide precipitation of H_2_S by ZnSO_4_. Thus, NIR-HS can be used for the selective detection of sulphide with minimum interference from other biological species.

### Detection of H_2_S in living cells

We thereafter assessed the potential utility of NIR-HS to monitor H_2_S in living MCF-7 cells. Prior to cell imaging, MTT assays were performed to evaluate the cytotoxicity of the probe. NIR-HS and compound 1 exhibited IC_50_ of 96.9 ± 3.2 μM and 99.4 ± 1.7 μM, respectively (Supplementary Figs S8 and S9). These results indicated that cells were variable after incubation with NIR-HS (5 μM) for 24 h.

**Figure 4:**
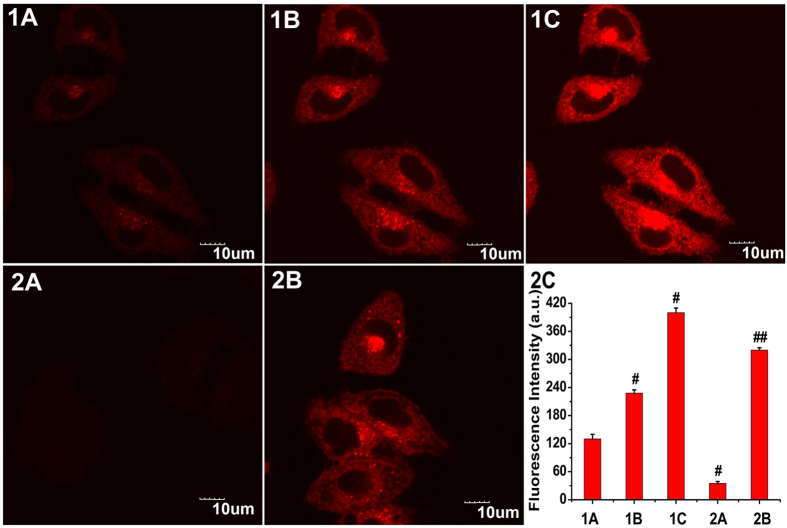
Confocal fluorescence imaging of exogenous sulphide in living MCF-7 cells using NIR-HS. Cells were incubated with NIR-HS (5 μM) alone for 10 min (**1A**). Cells in panel 1A were thereafter treated with Na_2_S (50 μM) for 10 min (**1B**) and 20 min (**1C**). Cells were pretreated with 1 mM ZnCl_2 _for 10 min, then incubated with NIR-HS (5 μM) for 10 min and Na_2_S (50 μM) for 20 min (**2A**). Cells were incubated with NIR-HS (5 μM) for 10 min and then further incubated with Na_2_S (25 μM) for 20 min (**2B**). Scale bars = 10 μm. The average fluorescence intensity of the above images (**2C**). Data are presented as the mean ± SD (*n* = 3). ^#^*p < *0.001 *vs.* (**1A**) column, ^##^*p < *0.001 *vs.* (**1C**) column.

MCF-7 cells incubated with the free probe (5 μM) showed relatively weak fluorescence emission (Fig. 4, panel 1A). In contrast, upon addition of Na_2_S (50 μM) to the above cells, a strong red fluorescence was observed (Fig. 4, panel 1B). The sulphide-induced increase of the fluorescence intensity in cells of panel 1B was finished after approximately 20 min (Fig. 4, panel 1C). Subsequently, cells were pretreated with ZnCl_2_ (an efficient eliminator of H_2_S). With the addition of Na_2_S (50 μM) to the ZnCl_2_-pretreated cells, no fluorescence intensity increases were observed (Fig. 4, panel 2A). The results indicated that the fluorescence change of NIR-HS in the cells arises from H_2_S. Moreover, the treatment of probe-loaded cells with Na_2_S (25 μM) yielded lower fluorescence emission (Fig. 4, panel 2B) compared to the fluorescence of the cells in panel 1C, implying that NIR-HS is capable of imaging different sulphide concentrations in living cells (Fig. 4, panel 2C).

**Figure 5:**
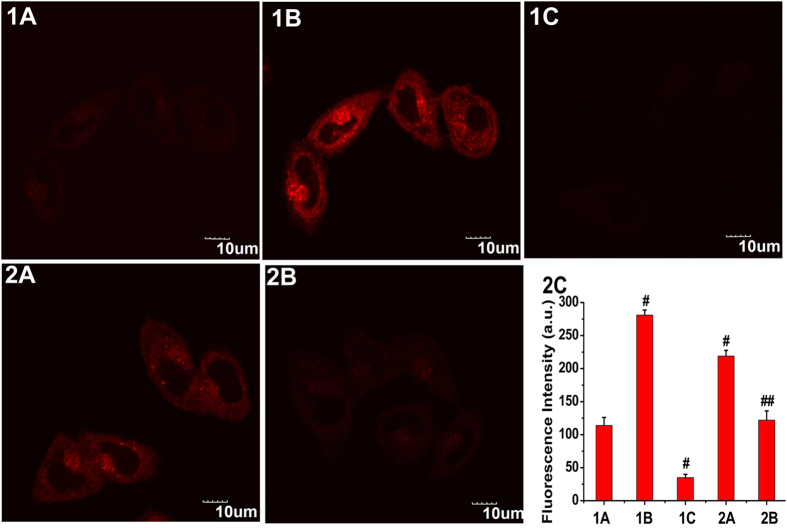
Confocal fluorescence imaging of endogenous H_2_S in living MCF-7 cells using NIR-HS. Cells were incubated with NIR-HS (5 μM) alone for 10 min (**1A**). Cells in panel 1A were thereafter treated with SNP (50 μM) for 20 min (**1B**). PPG-treated cells were incubated with NIR-HS (5 μM) for 10 min and then stimulated with SNP (50 μM) for 20 min (**1C**). Cells with CBS overexpression were incubated with NIR-HS (5 μM) for 10 min (**2A**). Cells transfected with empty vector were incubated with NIR-HS (5 μM) for 10 min (**2B**). Scale bars = 10 μm. The average fluorescence intensity of the above images (**2C**). Data are presented as the mean ± SD (*n* = 3). ^#^*p < *0.001 *vs.* (1A) column, ^##^*p < *0.001 *vs.* (**2A**) column.

Next, we tested the abilities of NIR-HS to visualize the endogenous H_2_S. MCF-7 cells express H_2_S-producing enzyme such as CSE^47^. NO could upregulate the CSE expression and stimulate the CSE activity, resulting in increased endogenous H_2_S level^48^. Therefore, SNP (Sodium Nitroprusside, a NO donor) was employed to induce the production of endogenous H_2_S in MCF-7 cells. The probe-loaded cells exhibited faint fluorescence emission without the addition of SNP (Fig. 5, panel 1A). After incubation of probe-treated cells with SNP (Fig. 5, panel 1B) for another 20 min, the fluorescence signal increased significantly, indicating the generation of endogenous H_2_S within the cells. Whereas the cells preincubated with DL-propargylglycine (PPG, an inhibitor for CSE^48^) provided almost no fluorescence enhancement (Fig. 5, panel 1C), demonstrating that the fluorescence change is triggered by endogenously generated H_2_S.

CSE and CBS are major enzymes for H_2_S production, and the overexpression of CBS or CSE could result in the elevation of endogenous H_2_S level^1^,^2^. We thus constructed the cells with CBS overexpression (Fig. 5, panel 2A). Cells transfected with empty vector (pCMV6) were set as control group (Fig. 5, panel 2B). As shown in Fig. 5, MCF-7 cells that were overexpressing CBS showed much stronger fluorescence (Fig. 5, panel 2A) than that from cells of the control group (Fig. 5, panel 2B), suggesting the increased endogenous level of H_2_S in CBS overexpressed cells. The western blot assay proved the overexpression of CBS in cells of panel 2A (Supplementary Fig. S12). We also quantified the fluorescence intensities of these cells, and found that the CBS overexpressed cells showed 2-fold enhanced fluorescence intensity compared to the control cells (Fig. 5, panel 2C). These results revealed the capability of NIR-HS to recognize endogenous H_2_S in living cells.

### Detection of H_2_S in living mice

**Figure 6:**
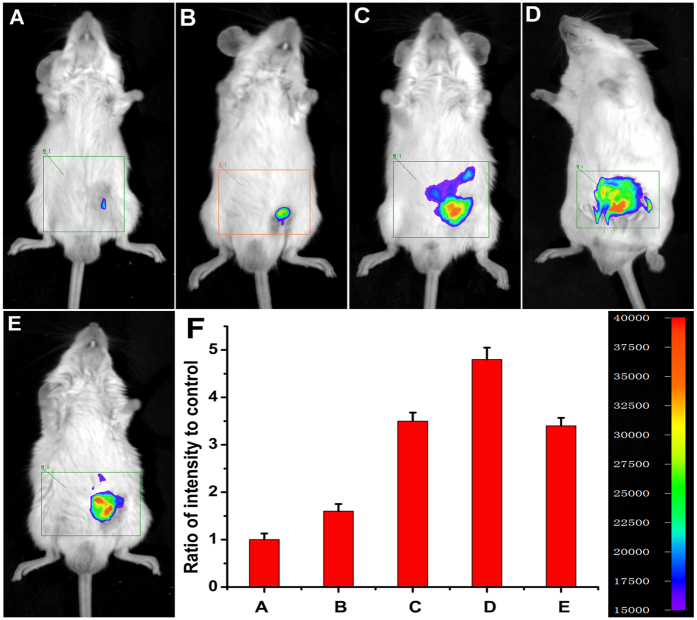
Representative fluorescence images of visualizing exogenous and endogenous H_2_S in living mice using NIR-HS. The mice were i.p. injected with the probe NIR-HS (50 μM, in 50 μL DMSO) as the control group (**A**). The mice were i.p. injected with the probe NIR-HS (50 μM, in 50 μL DMSO), followed by i.p. injection of 1 equiv. Na_2_S (50 μM, in 100 μL saline) (**B**). The mice were i.p. injected with the probe NIR-HS (50 μM, in 50 μL DMSO), followed by i.p. injection of 5 equiv. Na_2_S (250 μM, in 100 μL saline) (**C**). The mice were i.p. injected with the probe NIR-HS (50 μM, in 50 μL DMSO), followed by i.p. injection of 10 equiv. Na_2_S (500 μM, in 100 μL saline) (**D**). The mice were i.p. injected with SNP (200 μM, in 100 μL saline)), followed by i.p. injection of NIR-HS (50 μM, in 50 μL DMSO) (**E**). Quantification of the fluorescence emission intensities from the abdominal area of the mice of the above groups (**F**). Images were taken after incubation for 20 min. Data are presented as the mean ± SD (*n* = 3).

**Figure 7:**
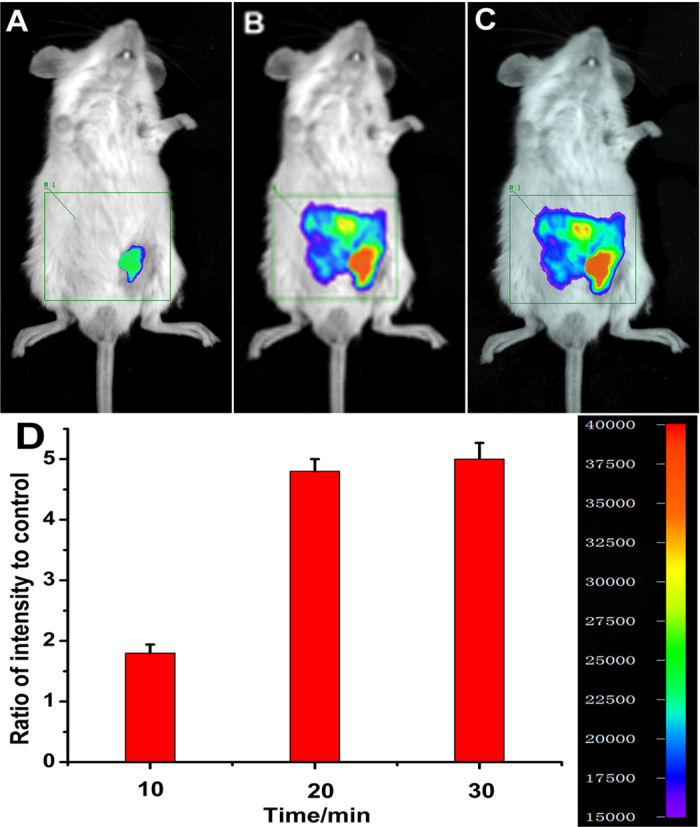
Representative fluorescence images of visualizing H_2_S levels at different times in living mice using NIR-HS. The mice were i.p. injected with the probe NIR-HS (50 μM, in 50 μL DMSO), followed by i.p. injection of 10 equiv. Na_2_S (500 μM, in 100 μL saline). Images were taken after incubation of Na_2_S at: 10 min (**A**); 20 min (**B**); 30 min (**C**). Quantification of the fluorescence emission intensities from the abdominal area of the mice of the above groups (**D**). Data are presented as the mean ± SD (*n* = 3).

The prominent NIR features of NIR-HS render the probe highly favorable for fluorescence imaging of H_2_S in living animals. Inspired by these data, we further examined the suitability of the sensor to visualize exogenous and endogenous H_2_S in living mice. Kunming mice were divided into several groups. The mice were given i.p. injection of DMSO as the negative control group (Supplementary Fig. S13, panel A), and the mice were given i.p. injection of free probe as the probe-loaded group (Fig. 6, panels A). One group were pretreated with ZnCl_2_, and then injected with free probe (Supplementary Fig. S13, panel B). The other three groups were injected with different amounts of Na_2_S (1, 5 and 10 equiv.) after i.p. injection of probe (Fig. 6, panels B, C and D). The last group were given i.p. injection of SNP and followed by i.p. injection with the probe. The mice were imaged using a Night OWL IILB 983 small animal *in vivo* imaging system. The fluorescent images showed almost no background fluorescence in the negative control group (Supplementary Fig. S13, panel A; panel C, R = 0.12 in column A), and weak fluorescence in the probe-loaded group (Fig. 6, panel A; panel F, R = 1.0 in column A), which suggests weak fluorescence signals in probe-loaded mice may be caused by endogenous H_2_S. To confirm this assumption, we pretreated (i.p. injection) another group of mice with ZnCl_2_ (an efficient eliminator of H_2_S). After 10 min of ZnCl_2_-treatment, the mice were given i.p. injection of free probe. Compared with the free probe-loaded mice, the fluorescence of ZnCl_2_-treated group is remarkably weakened (Supplementary Fig. S13, panel B; panel C, R = 0.18 in column B), indicating that the weak fluorescence in probe-loaded mice is triggered by physiological concentration of endogenous H_2_S. The mice treated with both Na_2_S (1, 5 and 10 equiv.) and the probe displayed much higher fluorescence (Fig. 6, panels B, C and D) than the mice treated with only the probe, which demonstrate that NIR-HS could respond to exogenous sulphide in mice. Moreover, the mice injected with SNP (Sodium Nitroprusside, a NO donor, could induce the production of endogenous H_2_S) and probe showed a maked elevation in the fluorescence intensities from the abdominal area of the mice (Fig. 6, panel E, R = 3.4 in column E), indicating that NIR-HS was sensitive enough to detect endogenous H_2_S in living mice. Importantly, the fluorescence intensities from the abdominal area of the mice were quantified, and the data showed that the fluorescence intensities triggered by Na_2_S were concentration-dependent (R = 1.0 in column A, R = 1.6 in column B, R = 3.5 in column C, R = 4.8 in column D) (Fig. 6, panel F). Figure 7 demonstrated that the fluorescence intensities became strong gradually within 20 min, consistent with the results of titrating the probe with Na_2_S at different time in PBS buffer (Supplementary Fig. S2). These experiments suggested that NIR-HS is suitable for monitoring exogenous and endogenous H_2_S in living mice.

Recently, the development of fluorescent probes for H_2_S *in vivo* is of high interest. A few fluorescent probes have been successfully discovered for imaging of H_2_S in living animals, such as mice^49^,^50^,^51^, zebrafish^52^,^53^,^54^,^55^ and Caenorhabditis elegans^56^,^57^, *et al.* In addition to fluorescent probes, luminescent probe and chemiluminescent probe have been applied to determining H_2_S in living mice^58^,^59^. Despite these progresses, the NIR fluorescence imaging of endogenous H_2_S *in vivo* is still highly desirable. Wallace *et al.* utilized fluorescent probe SF5 to investigate the regulation of leukocyte H_2_S synthesis *in vivo*^44^. However, probe SF5 emitted around 520 nm, the visible-light range limited its application for *in vivo* imaging due to the interference of background autofluorescence. Lu *et al.* prepared a novel bioluminescence probe for detection of endogenous H_2_S in nude mice^45^. Nevertheless, for this bioluminescence probe, Cys at 15 μM triggered weak bioluminescence. It is well known that the concentrations of Cys in cells/tissue are much higher compared to the concentrations of endogenous H_2_S, and small response induced by Cys may interference the detection of H_2_S. Moreover, the H_2_S reaction site of this bioluminescence probe was azide group. The azide-containing H_2_S probes could undergo photoactivation under continuous excitation^60^, rendering them unsuitable for *in vivo* imaging. Thus, NIR probes with high sensitivity, good selectivity and favourable properties to monitor endogenous H_2_S *in vivo* are highly needed. Our probe NIR-HS is more suitable for biological imaging endogenous H_2_S in living mice.

Taken together, we have prepared a novel fluorescent probe NIR-HS for H_2_S detection in living cells and mice. Advantages of this H_2_S-specific probe include emission in the NIR region, a low detection limit, high sensitivity, good selectivity and low cytotoxicity. This probe not only enables fluorescence imaging of endogenous H_2_S induced by SNP in living cells, but also detects endogenous H_2_S generated in cells overexpressing cystathionine β-synthase (CBS). The probe was also successfully applied to visualizing both the exogenous and endogenous H_2_S in living mice. Probe NIR-HS shows the potential to be used as a valuable research tool in studying biological roles of H_2_S. We are currently pursuing other strategies to develop more sensitive and specific fluorescent sensors for monitoring H_2_S in living animails, as well as the H_2_S-related medical studies.

## Methods

### Fluorometric analysis

All fluorescence measurements were conducted at room temperature on a Hitachi F4600 Fluorescence Spectrophotometer. The probe solution (CH_3_CN) was added to a quartz cuvette. With the probe diluted to 10 μM with 20 mM PBS buffer, Na_2_S was added (Na_2_S·9H_2_O serving as the H_2_S source in all experiments). The resulting solution was then incubated for 20 min. The samples were excited at 670 nm with the excitation and emission slit widths set at 5 nm and 10 nm, respectively. The emission spectrum was scanned from 690 nm to 850 nm at a velocity of 1200 nm/min. The photomultiplier voltage was set at 1000 V. Data are presented as the mean ± SD (*n* = 3).

### Cell culture and confocal fluorescence imaging

The MCF-7 cells were grown up in DMEM media supplemented with 10% (v/v) FBS (foetal bovine serum) and penicillin/streptomycin (100 μg/mL) at 37 °C in a 5% CO_2_ incubator. Cells were permitted to grow to 80% confluence before harvesting and transferring to a coverglass (Lab-Tek^®^ II Chambered Coverglass, NaleNunc, Naperville, USA). A final concentration of 5 μM NIR-HS (1.0 mM stock solution in CH_3_CN) was added to the cell media and incubated at the previous conditions for 10 min. For exogenous sulphide imaging, the cells were thrice rinsed with PBS solution (pH = 7.4) to remove excess NIR-HS, which was followed by the addition of Na_2_S (20 mM stock solution in DI H_2_O, final concentration 25 μM or 50 μM) for incubation 10 min or 20 min at 37 °C. For endogenous sulphide imaging, cells were pretreated with NIR-HS (5 μM) for 10 min and then stimulated with SNP (sodium nitroprusside, 20 mM stock solution in DI H_2_O, final concentration 50 μM) for 20 min. In addition, cells with CBS overexpression were incubated with NIR-HS (5 μM) for 10 min. All the cells were thrice rinsed with PBS buffer prior to imaging. Confocal fluorescence imaging was performed on an Olympus FV1000 confocal laser scanning microscope with ×60 oil objectives. The excitation wavelength was 635 nm. The fluorescence images (660 nm-760 nm) were obtained at 1024 × 1024 pixels, and were analysed with Olympus software (FV10-ASW). All data are expressed as the mean ± SD (*n* = 3).

### Animals and administration

Adult male Kunming mice weighing 20–25 g were provided by the Experimental Animal Centre of Xuzhou Medical College. All of the experiments were performed in compliance with the Chinese legislation on the use and care of laboratory animals and were approved by the Institutional Animal Care and Use of Xuzhou Medical College. The animals were housed in a room with regulated temperature (22 ± 2 °C) and humidity (50 ± 10%) on a 12 h light/dark cycle; the animals had *ad libitum* access to standard commercial animal feed and pure water. Mice were acclimatised for 1 week prior to the experiment.

### Fluorescent imaging in living mice

The mice were anesthetized by i.p. injection of 10% chloral hydrate (0.04 mL/10g), and their abdominal fur was removed. The mice were random selected and divided into several groups. Subsequently, one group were given i.p. injection of DMSO (50 μL) as the negative control group, and the mice were given i.p. injection of free probe (50 μM, in 50 μL DMSO) as the probe-loaded group. One group were pretreated with ZnCl_2_ (10 mM, in 100 μL saline), and then injected with free probe. The other three groups were i.p. injected with the probe NIR-HS (50 μM, in 50 μL DMSO), and followed by i.p. injection with different amount of Na_2_S (50 μM, 250 μM and 500 μM in saline). The last group were i.p. injected with SNP (200 μM, in 100 μL saline), followed by i.p. injection of NIR-HS (50 μM, in 50 μL DMSO). After 20 min, the mice were then imaged by using a Night OWL IILB 983 small animal *in vivo* imaging system, with an excitation filter of 670 nm and an emission filter of 690 nm–740 nm. For the time-dependent experiment, the mice were given i.p. injection of 10 equiv. Na_2_S (500 μM, in 100 μL saline) after the same disposal of the control mice. Images were then taken at different times (10, 20, and 30 min).

### Western blot

For western blot analysis, the cells were washed with cold PBS and lysed with RIPAa buffer (1% Triton X-100, 1% deoxycholate, 0.1% SDS) containing protease inhibitors (1mM PMSF, 20mM NaF, 1mM NaVO_3_). Protein concentrations were determined using BCA protein assay kits. The cells samples were separated by 10% SDS-polyacrylamide gel electrophoresis (SDS-PAGE) and then transferred onto nitrocellulose membranes (Amersham Pharmacia Biotech). The membranes were blocked with 5% skim milk powder in a washing buffer (Tris-buffered saline containing 0.05% (v/v) Tween 20) for 2 h at 25 °C and subsequently incubated overnight with the primary antibodies specific for CBS (1:1000) and β-actin(1:1000). Each membrane was thrice rinsed for 15 min and incubated with either alkaline phosphatase-conjugated secondary antibodies (1:1000, Goat anti-Rabbit IgG antibody) or alkaline phosphatase-conjugated secondary antibodies (1:1000, Horse anti-MouseIgG antibody), which was followed by visualization by BCIP/NBT alkaline phosphatase colour development kits. Protein bands were scanned and quantified by densitometric analysis using ImageJ version 1.34 s software.

### Construction of CBS overexpressing cells

The cDNA clones for human CBS were purchased from Origene (lot no.: RC201755). MCF-7 cells were grown to 90% confluence before being transiently transfected with pCMV6-control and pCMV6-CBS expression plasmids using Lipofectamine 2000 (Invitrogen, Shanghai, China) according to the manufacturer’s instructions. Six hours after transfection, the medium containing transfection reagents was removed and incubated in fresh medium. The CBS overexpressed cells were harvested for subsequent experiments.

### Statistical analyses

All statistical analyses were performed using SPSS software, version 16.0 (SPSS Inc., Chicago, IL, USA). Values are expressed as the mean ± SD (standard deviation of the mean). The data were analysed with one-way analysis of variance (ANOVA). Statistical significance was set at *p* < 0.05.

## Additional Information

**How to cite this article**: Zhang, L. *et al.* A highly selective and sensitive near-infrared fluorescent probe for imaging of hydrogen sulphide in living cells and mice. *Sci. Rep.* **6**, 18868; doi: 10.1038/srep18868 (2016).

## Supplementary Material

Supplementary Information
