# Supplementary material for: A highly selective and sensitive near-infrared fluorescent probe for imaging of hydrogen sulphide in living cells and mice
Source: Sci Rep. 2016 Jan 8;6:18868. doi: 10.1038/srep18868 (PMC4705538; doi:10.1038/srep18868)
Supplement: Supplementary Information [file srep18868-s1.pdf]

Electronic Supplementary Information for

A highly selective and sensitive near-infrared fluorescent probe for imaging of hydrogen sulfide in living cells and mice

Ling Zhang[1,2]*, Xi Emily Zheng[4*], Fang Zou[2], Yanguo Shang[2], Wenqi Meng[2], En Lai[1], Zhichen Xu[1], Yi Liu[2] & Jing Zhao[1,3]

[1]School of Chemistry and Chemical Engineering, Nanjing University, Nanjing, 210093, China.

[2]Jiangsu Key Laboratory of New Drug Research and Clinical Pharmacy, School of Pharmacy, Xuzhou Medical College, Xuzhou, 221002, China.

[3]Guangdong Key Lab of Nano-Micro Material Research, School of Chemical Biology and Biotechnology, Peking University Shenzhen Graduate School, Shenzhen, 518055, China.

[4]Department of Gastroenterology, Nanjing Drum Tower Hospital, The Affiliated Hospital of Nanjing University Medical School , Nanjing, 210029, China.

Table of contents

General information
Chemicals and media
Synthesis and Characterisation of compounds
Evidence of mechanism detection
Quantum yields
Determination of the detection limit
Preparation of the test solution
Absorption analyses
MTT Assay
References
Figure S1. Fluorescence spectra and absorption spectra of compound 1, NIR-HS and Na_2S + NIR-HS
Figure S2. Time profile of NIR-HS toward sulphide
Figure S3. Effects of pH on NIR-HS in PBS buffer
Figure S4. Effects of pH on compound 1 in PBS buffer
Figure S5. Selectivity of NIR-HS to various thiols
Figure S6. Selectivity of NIR-HS to various amino acids
Figure S7. Selectivity of NIR-HS to other species
Figure S8. Cell viability of MCF-7 cells in the presence of NIR-HS
Figure S9. Cell viability of MCF-7 cells in the presence of compound 1
Figure S10. The corresponding bright images of cells
Figure S11. The corresponding bright images of cells
Figure S12. CBS protein expression levels of cells
Figure S13. Fluorescence images in living mice
Figure S14-17. NMR and HRMS spectrum of compounds

General Information

Thin layer chromatography was performed on silica gel 60 F_{254} plates (250 μm) and column chromatography was conducted over silica gel (300-400 mesh). Visualization of the developed chromatogram was accomplished by a UV lamp. Nuclear magnetic resonance (NMR) spectra were acquired on Bruker DRX-400 operated at 125/100 MHz for ^{1}H NMR and ^{13}C NMR, respectively, residual protio solvent signals serving as internal criteria for calibration purposes. Data for ^{1}H NMR are reported as follows: chemical shift (ppm), multiplicity (s, singlet; d, doublet; t, triplet; q, quartet; m, multiplet), integration, coupling constant (Hz). High-Resolution Mass was performed by Mass Spectrometry. All fluorescence measurements were recorded on a Hitachi F4600 Fluorescence Spectrophotometer. The pH measurements were performed on a Mettler-Toledo Delta 320 pH meter. All fluorescence imaging experiments were conducted on a FV1000 confocal laser scanning microscope (Olympus, Japan). The in *vivo* imaging was carried out using a Night OWL Ⅱ LB 983 small animal in *vivo* imaging system.

Chemicals and media

Unless noted otherwise, reagents and solvents were obtained from commercial suppliers and employed without further purification: $Na_2S\cdot 9H_2O$ (≥ 99.99%), DMEM media, fetal bovine serum (FBS), penicillin (100 μg/mL) and streptomycin (100 μg/mL) (Life Technologies, CA, USA); Pierce BCA Protein Assay Kit (Thermo Scientific, Rockford, IL, USA); MCF-7 cells (the Committee on type Culture Collection of Chinese Academy of Sciences); CBS (Santa Cruz Biotechnology, Santa Cruz, CA, USA); β-actin (ZSGB-BIO, Nanjing, China); alkaline phosphatase-conjugated antibodies (1:1000, ZSGB-BIO, Jiangsu, China); BCIP/NBT alkaline phosphatase colour development Kits (Beyotime Institute of Biotechnology, Jiangsu, China); cDNA clones (lot no.: RC201755) for human CBS (Origene, Beijing, China); Lipofectamine 2000 (Invitrogen, Shanghai, China).

Synthesis and Characterisation of compounds

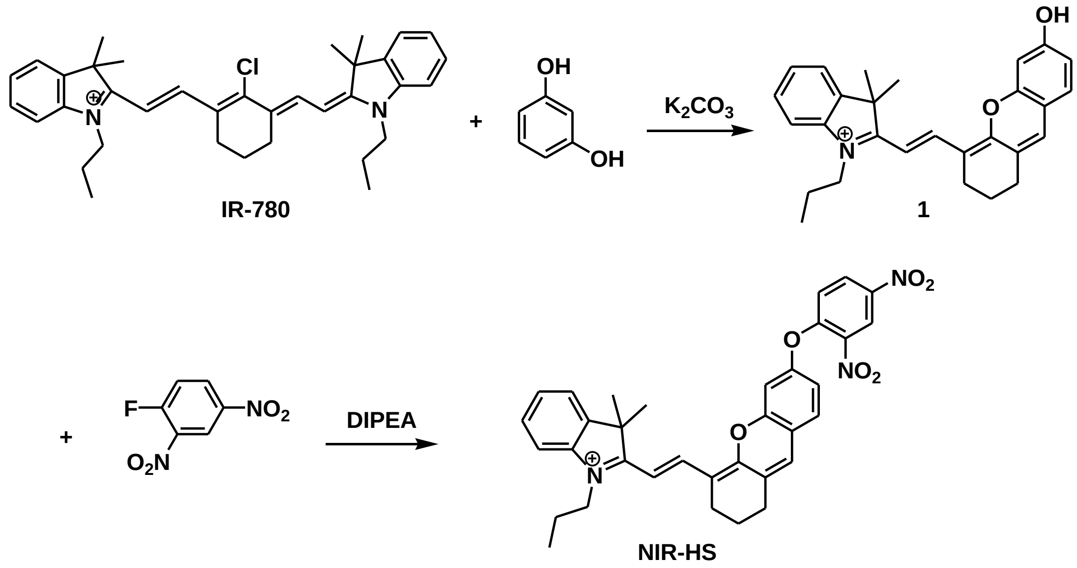

Synthesis of Compound 1. Compound 1 was synthesized according to the method reported by Zhu *et al*[1]. Resorcin (248 mg, 2.3 mmol) and K_2CO_3 (311 mg, 2.3 mmol) were placed in a flask containing CH_3CN (5 mL), and the mixture was stirred at room temperature under nitrogen atmosphere for 10 min. Then IR-780 iodide (600 mg, 0.9 mmol) in CH_3CN (3 mL) was added and the mixture was heated at 50 °C for 2 h. The solvent was evaporated and the crude product was purified by column chromatography on SiO_2 to give the purified product,

a blue-green solid (220 mg, yield 59.3%). TLC (silica, CH_2Cl_2: CH_3OH, 10:1 v/v): R_f = 0.4; ^1H NMR (400 MHz, $CDCl_3$): δ 8.50 (d, *J* = 16.0 Hz, 1H), 7.39-7.43 (m, 3H), 7.30 (d, *J* = 8.8 Hz, 1H), 7.23-7.27 (m, 2H), 7.14(d, *J* = 7.6 Hz, 1H), 7.06 (dd, *J*= 2.0, 8.8 Hz, 1H), 6.07(d, *J* = 14.4 Hz, 1H), 4.11(t, *J* = 7.6 Hz, 2H), 2.76 (t, *J* = 6.0 Hz, 2H), 2.70 (t, *J* = 6.4 Hz, 2H), 1.91-1.96 (m, 4H), 1.77 (s, 6H), 1.10 (t, *J* =7.6 Hz, 3H); ^{13}C NMR (100 MHz, $CDCl_3$): δ 178.7, 167.6, 161.0, 158.5, 143.2, 140.0, 139.9, 135.2, 129.7, 128.3, 125.3, 123.2, 121.9, 118.3, 115.9, 115.6, 108.9, 103.5, 95.6, 48.1, 45.3, 28.7, 28.2, 24.5, 21.3, 20.1, 11.5; HRMS: m/z calcd for compound 1 ($C_{28}H_{30}NO_2$, M^+) 412.2271; found, 412.2272.

Synthesis of Compound NIR-HS. Compound 1 (220 mg, 0.53 mmol), N,N-Diisopropylethylamine (68 mg, 0.53mmol) and 2,4-dinitrofluorobenzene (119 mg, 0.64 mmol) were dissolved in dry CH_2Cl_2(8 mL). The mixture was stirred at room temperature. After overnight reaction, the solvent was removed under reduced pressure. The resulting residue was purified by a silica gel column to afford compound NIR-HS as a blue solid (153 mg, yield: 50.0%). TLC (silica, EtOAc: CH_3OH, 8:1 v/v): R_f = 0.5; ^1H NMR (400 MHz, $CDCl_3$): δ 8.91 (d, *J* = 2.8 Hz, 1H), 8.64-8.68 (m, 1H), 8.48-8.51 (m, 1H), 7.44-7.54 (m, 5H), 7.35 (d, *J* = 9.2 Hz, 1H), 7.13-7.15 (m, 2H), 6.99 (dd , *J* = 8.4, 2.0 Hz, 1H), 6.83 (d, *J* = 15.2 Hz, 1H), 4.64-4.68 (m, 2H), 2.89-2.93 (m, 2H), 2.78-2.81 (m, 2H), 1.84 (s, 6H), 1.13 (t, *J* = 7.6 Hz, 3H). ^{13}C NMR (100 MHz, $CDCl_3$): 178.8, 159.7, 155.7, 154.5, 153.9, 146.4, 142.5, 142.2, 141.3, 139.9, 130.6, 129.6, 129.5, 129.4, 128.1, 122.7, 122.0, 120.5, 120.2, 116.8, 115.7, 113.4, 108.1, 106.6, 51.2, 48.3, 29.5, 28.2, 24.8, 21.6, 20.1, 11.6; HRMS: m/z calcd for NIR-HS ($C_{34}H_{32}N_3O_6$, M^+) 578.2286; found, 578.2237.

Evidence of mechanism detection

NIR-HS (60 mg, 0.1 mmol) was dissolved in CH_3CN (15 mL), followed by the addition of the solution of Na_2S• $9H_2O$ (240 mg, 1.0 mmol) in PBS buffer (15mL, 20 mM, pH = 7.4). The resultant mixture was stirred for 3 h at room temperature. Subsequently, EtOAc (3 x 10 mL) was added into the solution for extraction. The thiolysis product was characterised by HRMS and ^1H NMR, which were consistent with those of compound 1, hence the confirmation of the fluorescent product as compound 1.

Quantum Yields

Quantum yields were determined using fluorescein as a standard according to a published method[2]. For NIR-HS and fluorescein, the absorbance spectra were measured within an absorbance range of 0.01 to 0.1. The quantum yield was calculated according to the equation: $\Phi_{sample} = \Phi_{standard}\ (Grad_{sample}/Grad_{standard})(\eta^2_{sample}/\eta^2_{standard})$; where Φ is the quantum yield, $\Phi_{fluorescein}$ = 0.79 in 0.1 M NaOH, Grad is the slope of the plot of absorbance versus integrated emission intensity, and η is the refractive index of the solvent.

Determination of the detection limit

The detection limit was calculated based on the method reported in the previous literature[3]. The fluorescence emission spectrum of NIR-HS without Na_2S was measured by 10 times and the standard deviation of blank measurement was obtained. Then the solution was treated with Na_2S of concentration from 0 to 100 μM. A linear regression curve was then achieved according to the fluorescence intensity in the range of Na_2S from 0 to 2 μM. The detection limit was calculated with the following equation: Detection limit = 3σ/k. Where σ is the standard deviation of blank measurements, k is the slope between the fluorescence intensity ratios versus Na_2S concentrations. The detection limit was 38 nM in PBS buffer.

Preparation of the test solution

NIR-HS stock solution preparation: NIR-HS (5.79 mg, 0.01 mmol) was dissolved into CH_3CN (10 mL) to get 1.0 mM stock solution.

Cys (L-Cysteine) stock solution preparation: Cys (24.2 mg, 0.2 mmol) was dissolved into DI H_2O (10 mL) to get 20.0 mM stock solution, which was then diluted to 1.0 mM and 100 μM solution for general use.
Hcy (Homocysteine) stock solution preparation: Hcy (27.0 mg, 0.2 mmol) was dissolved into DI H_2O (10 mL) to get 20.0 mM stock solution, which was then diluted to 1.0 mM and 100 μM solution for general use.
GSH (Glutathione) stock solution preparation: GSH (61.5 mg, 0.2 mmol) was dissolved into DI H_2O (10 mL) to get 20.0 mM stock solution, which was then diluted to 1.0 mM and 100 μM solution for general use.
Na_2S stock solution preparation[4]: 5 mg EDTA was dissolved in 10 mL DI H_2O in a 25 mL Schlenk tube. The solution was purged vigorously with nitrogen for 15 min. Then 48 mg sodium sulfide ($Na_2S{\cdot}9H_2O$) was dissolved in the solution under nitrogen. The resulting solution was 20 mM Na_2S, which was then diluted to 1.0 mM-100 μM stock solution for general use.
Stock solutions of other biological analytes, including Ala, Glu, Trp, Met, Tyr, Leu, Val, Ser, Pro, Arg, Gly, Phe, His, Gln, Asn, Ile, Thr, KCl, $CaCl_2$, NaCl, $MgCl_2$, $ZnSO_4$, $FeCl_3$, NaH_2PO_4, H_2O_2, $\cdot OCl^-$, O_2^-, $\cdot OH$, ^tBuOOH, NO_2^-, NO, $Na_2S_2O_3$, $Na_2S_2O_5$, Na_2SO_4, $Na_2S_2O_4$, Na_2SO_3, KSCN, NADH, and Glucose, were prepared in DI H_2O. Superoxide radicals (O^{2-}) were generated according to the previous reported method[5]. $\cdot OH$ was generated by Fenton reaction between Fe^{II}(EDTA) and H_2O_2 quantitively[6]. NO is generated in form of 3-(Aminopropyl)-1-hydroxy-3-isopropyl-2-oxo-1-triazene (NOC-5, 50 μmol/ml).

Absorption analyses

Absorption spectra were recorded at room temperature on a Shimadzu PharmaSpec UV-2401PC UV-Visible spectrophotometer. The probe solution (CH_3CN) was added to a quartz cuvette. With the probe diluted to 10 μM with 20 mM PBS buffer, Na_2S was added. The resulting solution was incubated for 20 min prior to measurements (n = 3), with the mean ± SD expressed.

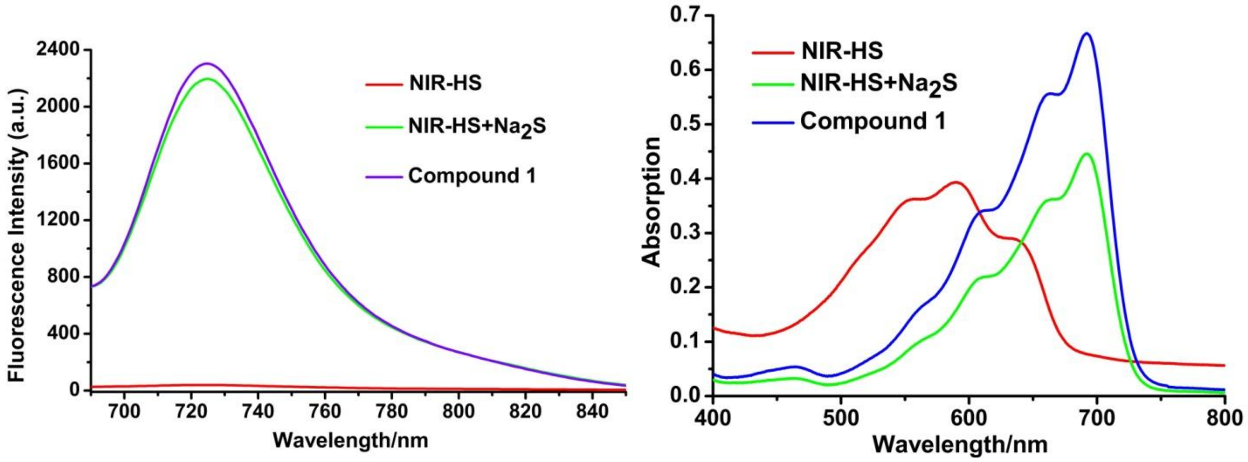

Figure S1 (A) Fluorescence spectra of compound 1, NIR-HS and Na_2S + NIR-HS in PBS buffer (20 mM, pH = 7.4, 5 % CH_3CN). (B) Absorption spectra of compound 1, NIR-HS and Na_2S + NIR-HS in PBS buffer (20 mM, pH = 7.4, 5 % CH_3CN).

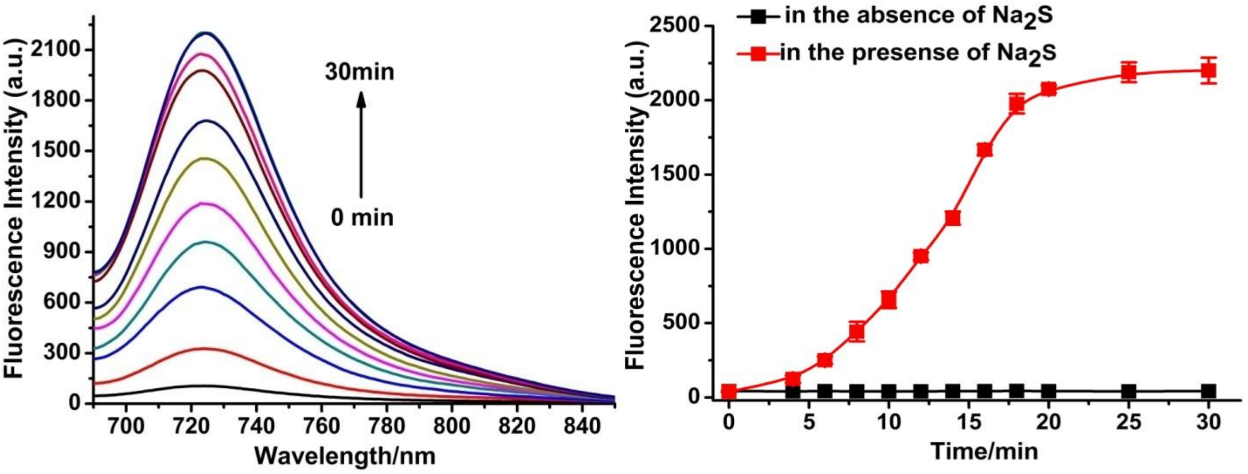

Figure S2 (A) Fluorescence spectra of NIR-HS (10 μM) with Na_2S (100 μM) in PBS buffer (20 mM, pH 7.4, 5 % CH_3CN) at 37°C for 0, 4, 6, 8, 10, 12, 14, 16, 18, 20, 25 and 30 min. (B) Time profile of NIR-HS (10 μM) toward Na_2S (100 μM) in PBS buffer (20 mM, pH 7.4, 5 % CH_3CN) at 37 ^{o}C for 0, 4, 6, 8, 10, 12, 14, 16, 18, 20, 25 and 30 min. Data are presented as the mean ± SD (n = 3).

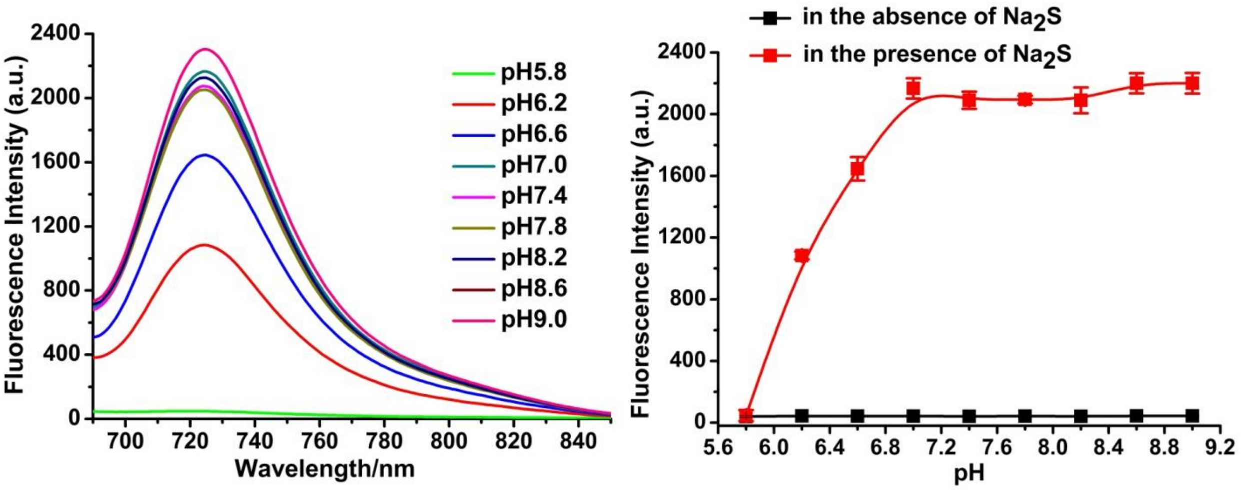

Figure S3 (A) Fluorescence spectra of NIR-HS (10 μM) with Na_2S (100 μM) in different pH buffer (20 mM, pH 5.8, 6.2, 6.6, 7.0, 7.4, 7.8, 8.2, 8.6, and 9.0, 5% CH_3CN) at 37 ^{o}C for 20 min. (B) Fluorescence responses of NIR-HS (10 μM) with Na_2S (100 μM) in different pH buffer (20 mM, pH 5.8, 6.2, 6.6, 7.0, 7.4, 7.8, 8.2, 8.6, and 9.0, 5% CH_3CN) at 37 ^{o}C for 20 min.

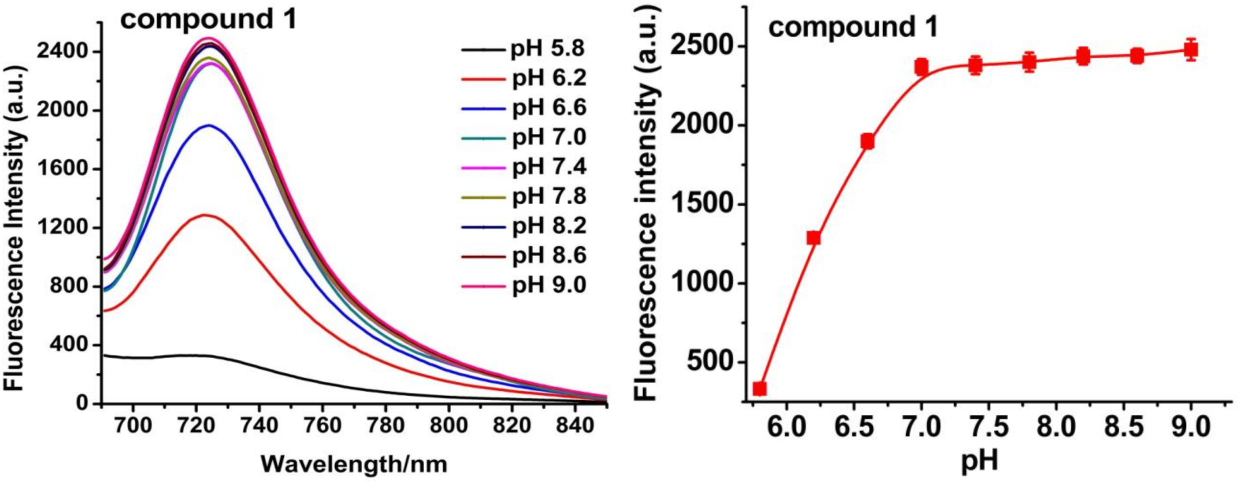

Figure S4 (A) Fluorescence spectra of compound **1** (10 μM) in different pH buffer (pH 5.8, 6.2, 6.6, 7.0, 7.4, 7.8, 8.2, 8.6, and 9.0, 5% CH_3CN) at 37 ^{o}C. (B) Fluorescence responses of compound **1** (10 μM) in different pH buffer (pH 5.8, 6.2, 6.6, 7.0, 7.4, 7.8, 8.2, 8.6, and 9.0, 5% CH_3CN) at 37 ^{o}C.

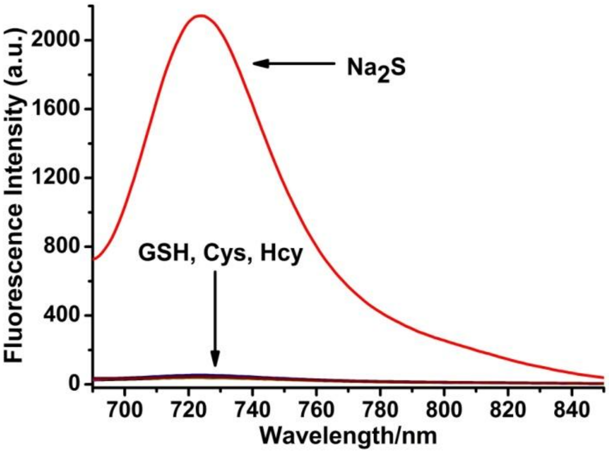

Figure S5 Fluorescence spectra of NIR-HS (10 μM) towards Na_2S (100 μM) and various biothiols (100 μM Hcy; 1 mM GSH; 100 μM Cys; 1 mM Cys; 10 mM GSH; 1 mM Hcy) after 20 min of incubation.

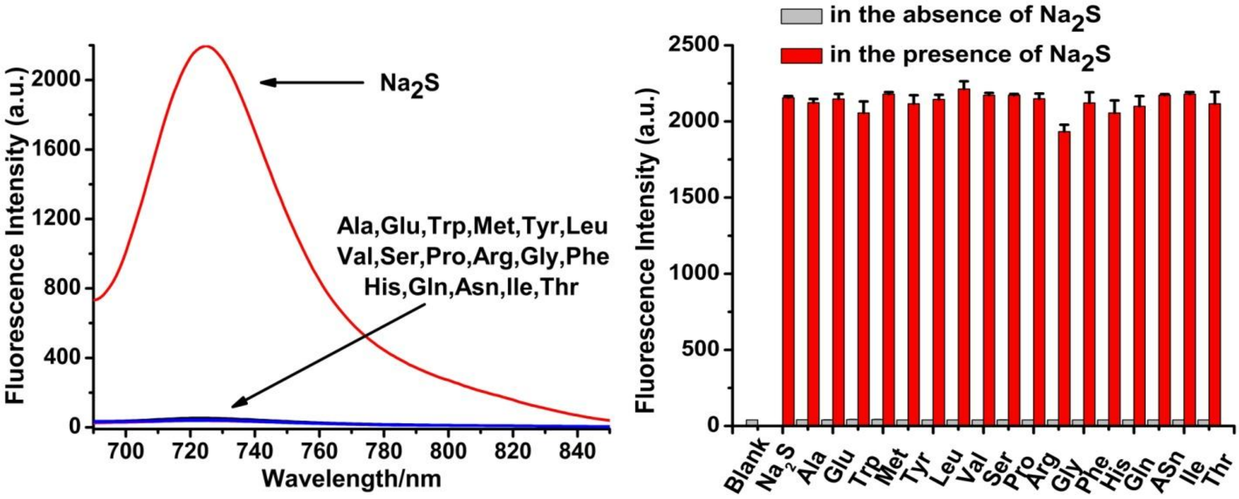

Figure S6 (A) Fluorescence spectra of NIR-HS (10 μM) with Na_2S (100 μM) and various amino acids (1 mM) after 20 min of incubation. (B) Fluorescence responses of NIR-HS (10 μM) with Na_2S (100 μM) and various amino acids (1 mM) after 20 min of incubation. Data are presented as the mean ± SD (*n* = 3).

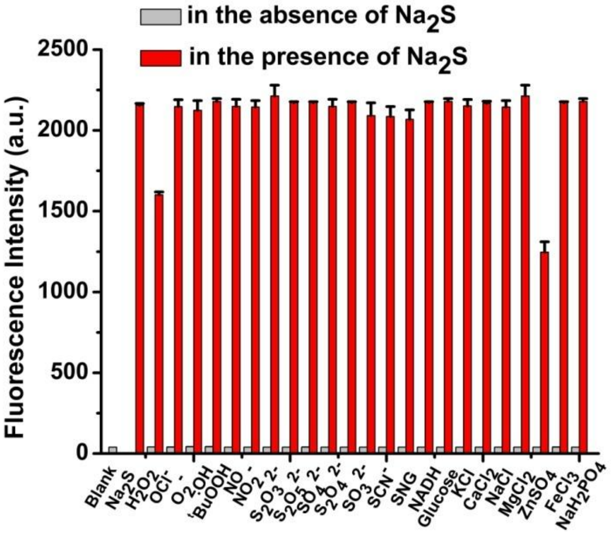

Figure S7 Fluorescence responses of NIR-HS (10 μM) towards Na_2S (100 μM), reactive oxygen species (H_2O_2, $\cdot OCl^-$, O_2^-, ·OH, ^{t}BuOOH, 1 mM), reactive nitrogen species (NO, NO_2^-, 1 mM), sulphur-containing inorganic ions ($S_2O_3^{2-}$, $S_2O_5^{2-}$, SO_4^{2-}, $S_2O_4^{2-}$, SO_3^{2-}, SCN^-, 1mM), reducing agents (NADH, Glucose) and inorganic salts (KCl, $CaCl_2$, NaCl, $MgCl_2$, $FeCl_3$, $ZnSO_4$, NaH_2PO_4, 1mM) and S-nitroso glutathione (SNG, 1 mM) after 20 min of incubation. Data are presented as the mean ± SD (*n* = 3).

MTT assay

Cell growth inhibitory effects of NIR-HS and compound 1 were measured using a colorimetric MTT assay kit (Sigma-Aldrich). MCF-7 cells were seeded in 96-well plates at a density of 50,000 cells/well and then maintained at 37 °C in a 5 % CO_2 incubator. The cells were incubated with different concentrations of NIR-HS and compound 1 for 24 h, respectively. Cells in culture medium without NIR-HS and compound 1 were used as control. After the incubation time, 20 μL of MTT dye (3-[4, 5-dimethylthiazol-2-yl]- 2, 5-diphenyl tetrazolium bromide, 5 mg/ml in phosphate buffered saline), was added to each well, and the plates were incubated for 4 h at 37 ℃. Then, the remaining MTT solution was removed, and 150 μL of DMSO was added to each well to dissolve the formazan crystals. The plate was shaken for 10 min and the absorbance was measured at 570 nm on a microplate reader (ELX808IU, Bio-tek Instruments Inc, USA). Each sample was performed in triplicate, and the entire experiment was repeated three times. Calculation of IC_{50} values was done according to Huber and Koella. IC_{50} of NIR-HS and compound 1 was calculated to be of 96.9 ± 3.2 μM and 99.4 ± 1.7 μM, respectively. The cell viability of NIR-HS and compound 1 (5 μM) at 0, 6, 12, 18 and 24 h further demonstrated that the NIR-HS and compound 1 were of low toxicity to cultured MCF-7 cells.

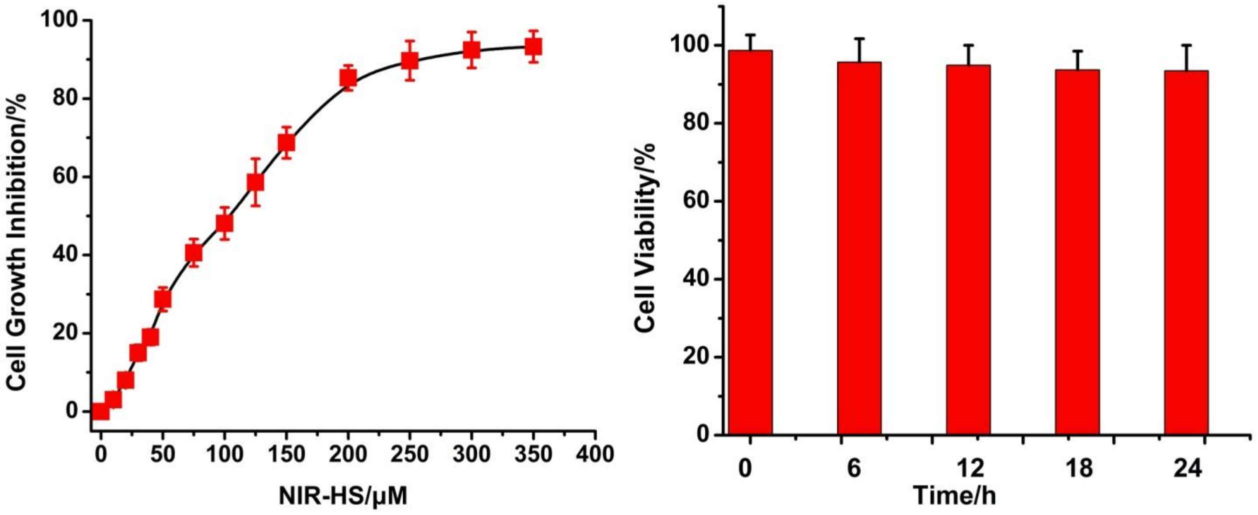

Figure S8 (A) The inhibitory effect of NIR-HS on cell growth in MCF-7 cells treated for 24 h. (B) Cell viability of NIR-HS (5 μM) at different times in MCF-7 cell. Data are presented as the mean ± SD (*n* = 3).

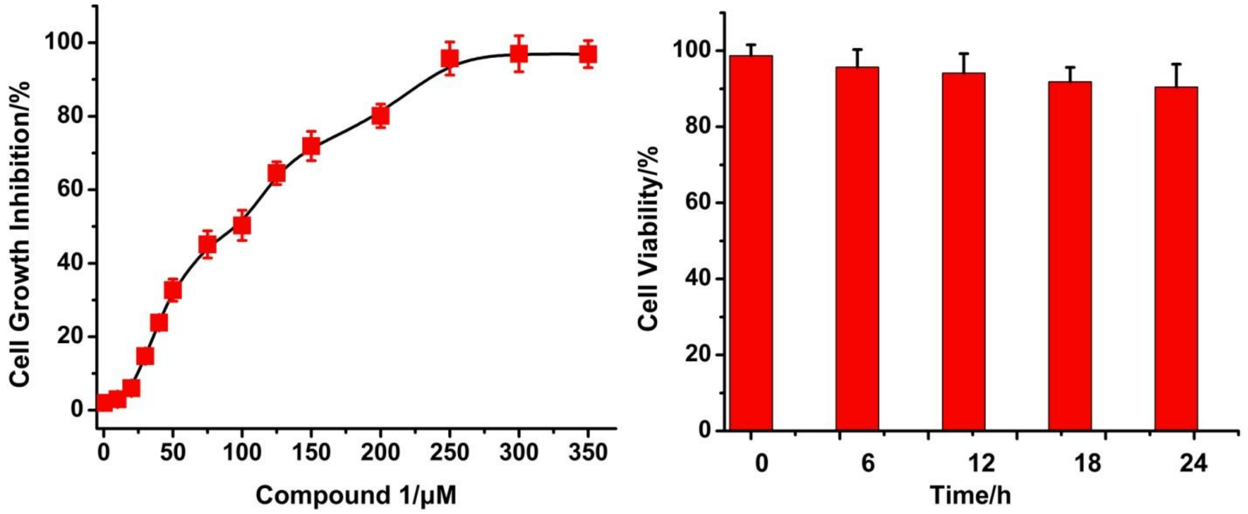

Figure S9 (A) The inhibitory effect of compound 1 on cell growth in MCF-7 cells treated for 24 h. (B) Cell viability of compound 1 (5 μM) at different times in MCF-7 cell. Data are presented as the mean ± SD (*n* = 3).

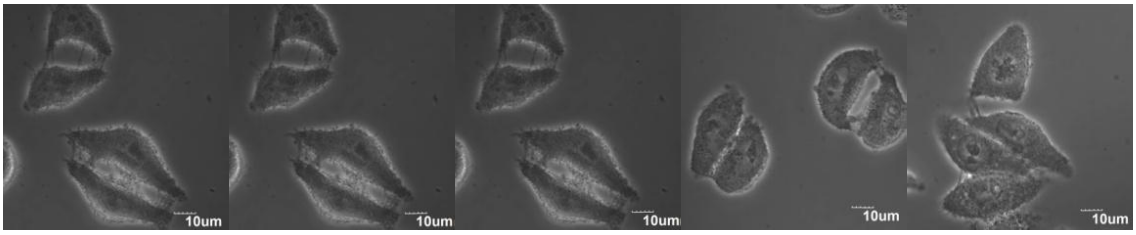

Figure S10 The corresponding bright images of Fig. 3, panels 1A, 1B, 1C, 2A and 2B.

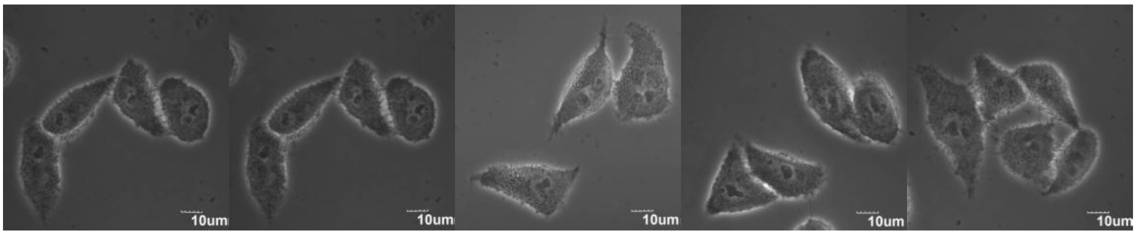

Figure S11 The corresponding bright images of Fig. 4, panels 1A, 1B, 1C, 2A and 2B .

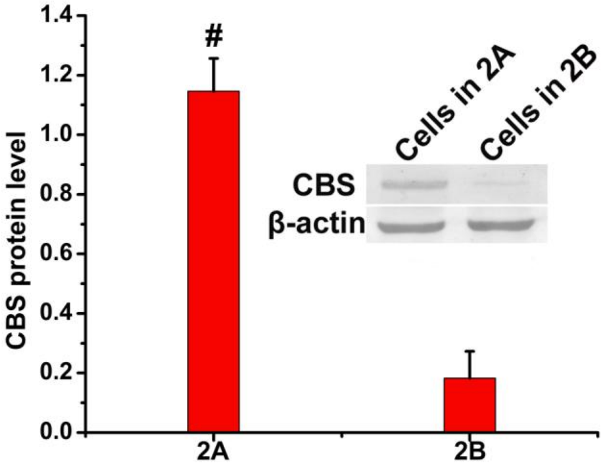

Figure S12 CBS protein expression levels of cells were analyzed by western blot assay. (2A) Cells were transfected with pCMV6-CBS expression plasmids. (2B) Cells were transfected with empty vector, pCMV6. Data are presented as the mean ± SEM (n = 3). $^{\#}p$<0.001 *vs.* 2B column.

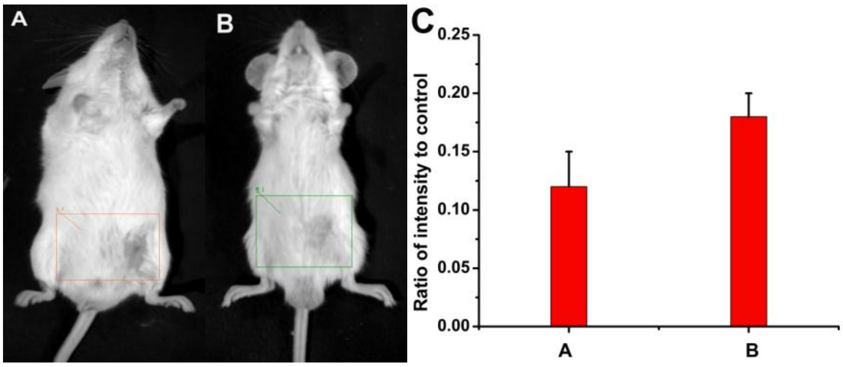

Figure S13 Fluorescence images in living mice. The mice were i.p. injected with DMSO (50 μL) as the negative control group (A). The mice were i.p. injected with the $ZnCl_2$ (10 mM, in 100 μL saline), after 10 min, the mice were i.p. injected with probe NIR-HS (50 μM, in 50 μL DMSO) (B). Quantification of the fluorescence emission intensities from the abdominal area of the mice of groups A and B (C). Data are presented as the mean ± SD (n = 3).

References

1. Yuan, L. *et al*. A unique approach to development of near-infrared fluorescent sensors for in vivo imaging. *J. Am. Chem. Soc.* **134,** 13510-13523 (2012).
2. Williams, A. T. R., Winfield, S. A. & Miller, J. N. Relative fluorescence quantum yields using a computer-controlled luminescence spectrometer. *Analyst*. **108,** 1067-1071 (1983).
3. Joshi, B. P., Park, J., Lee. W. I. & Lee, K. Ratiometric and turn-on monitoring for heavy and transition metal ions in aqueous solution with a fluorescent peptide sensor. *Talanta*. **78,** 903-909 (2009).
4. Qian, Y. *et al*. A Fluorescent Probe for rapid detection of hydrogen Sulfide in blood plasma and brain tissues in mice. *Chem. Sci*. **3,** 2920-2923 (2012).
5. Arudi, R., Allen, A. & Bielski, B. Some observations on the chemistry of KO_2-DMSO solutions. *FEBS. Lett.* **135,** 265-267 (1981).
6. Halliwell, B. & Gutteridge, J. M. Oxygen free radicals and iron in relation to biology and medicine: some problems and concepts. *Arch. Biochem. Biophys.* **246,** 501-514 (1986).

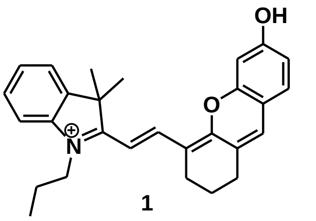
OH
O
N

1

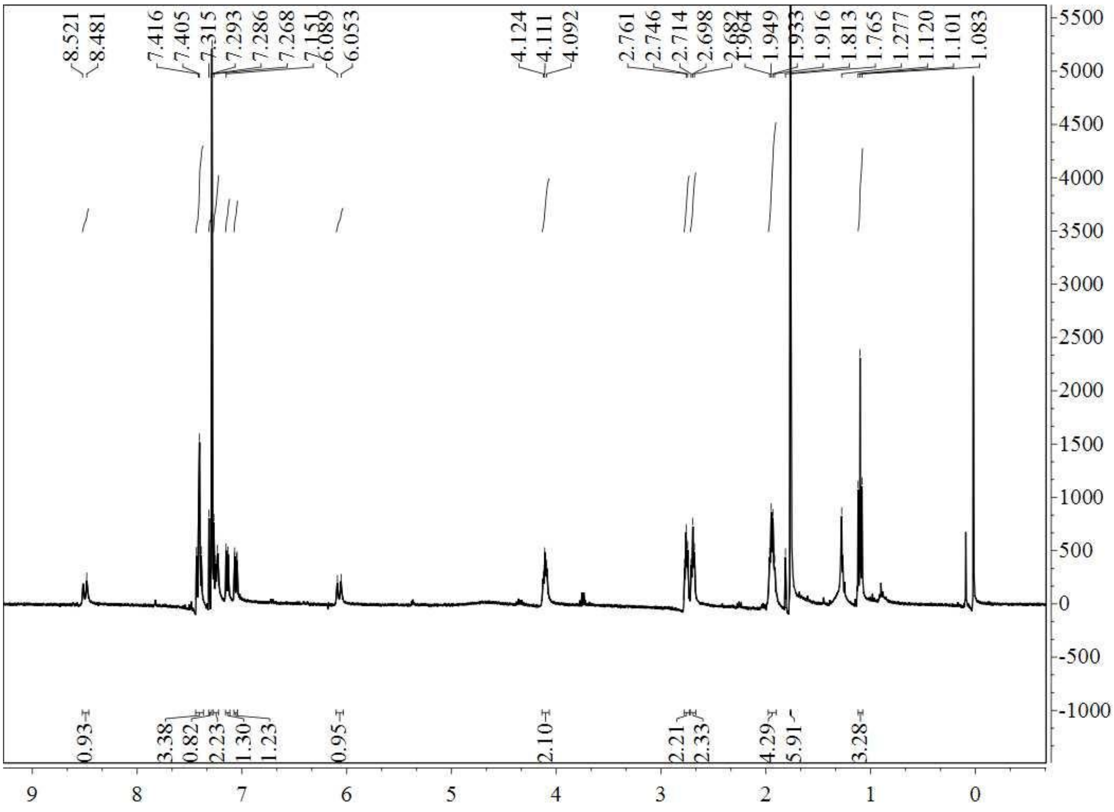
8.521
8.481
7.416
7.405
7.315
7.293
7.286
7.268
7.151
6.089
6.053
4.124
4.111
4.092
2.761
2.746
2.714
2.698
2.682
1.964
1.949
1.933
1.916
1.813
1.765
1.277
1.120
1.101
1.083
0.93
3.38
0.82
2.23
1.30
1.23
0.95
2.10
2.21
2.33
4.29
5.91
3.28
5500
5000
4500
4000
3500
3000
2500
2000
1500
1000
500
0
-500
-1000
9
8
7
6
5
4
3
2
1
0

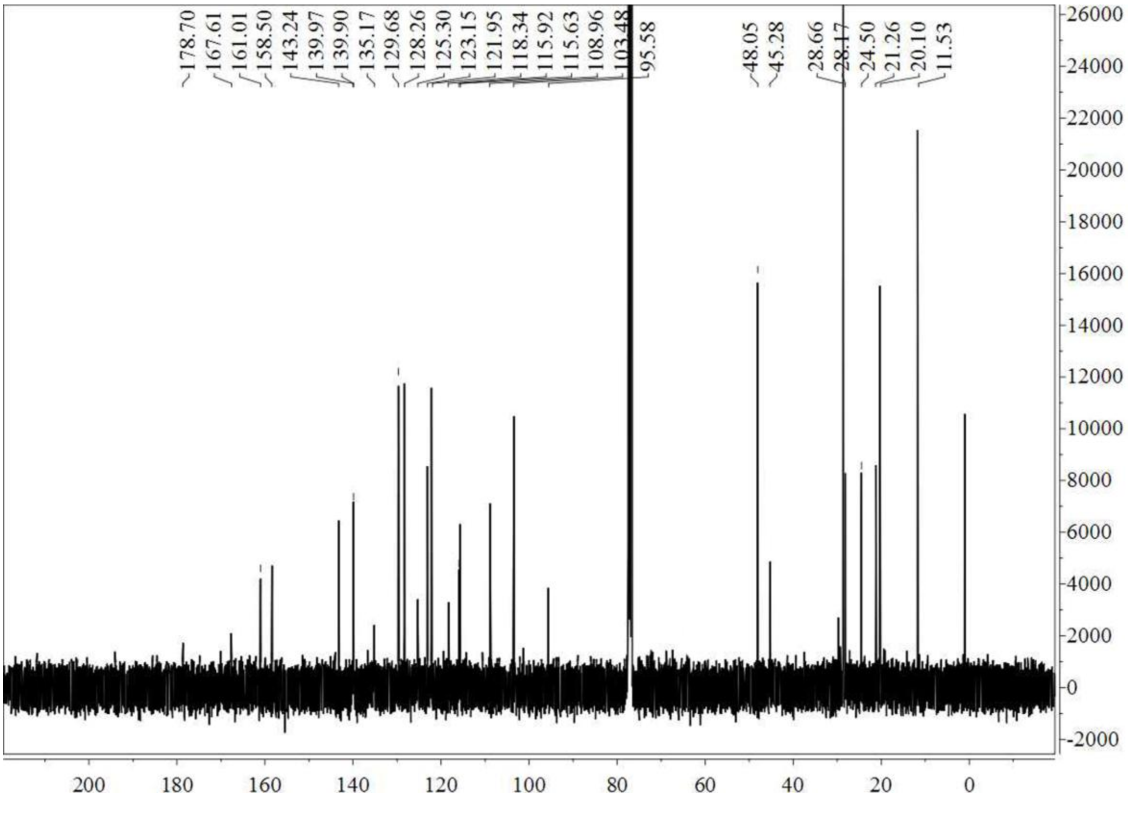
178.70
167.61
161.01
158.50
143.24
139.97
139.90
135.17
129.68
128.26
125.30
123.15
121.95
118.34
115.92
115.63
108.96
103.48
95.58
48.05
45.28
28.66
28.17
24.50
21.26
20.10
11.53
26000
24000
22000
20000
18000
16000
14000
12000
10000
8000
6000
4000
2000
0
-2000
200
180
160
140
120
100
80
60
40
20
0

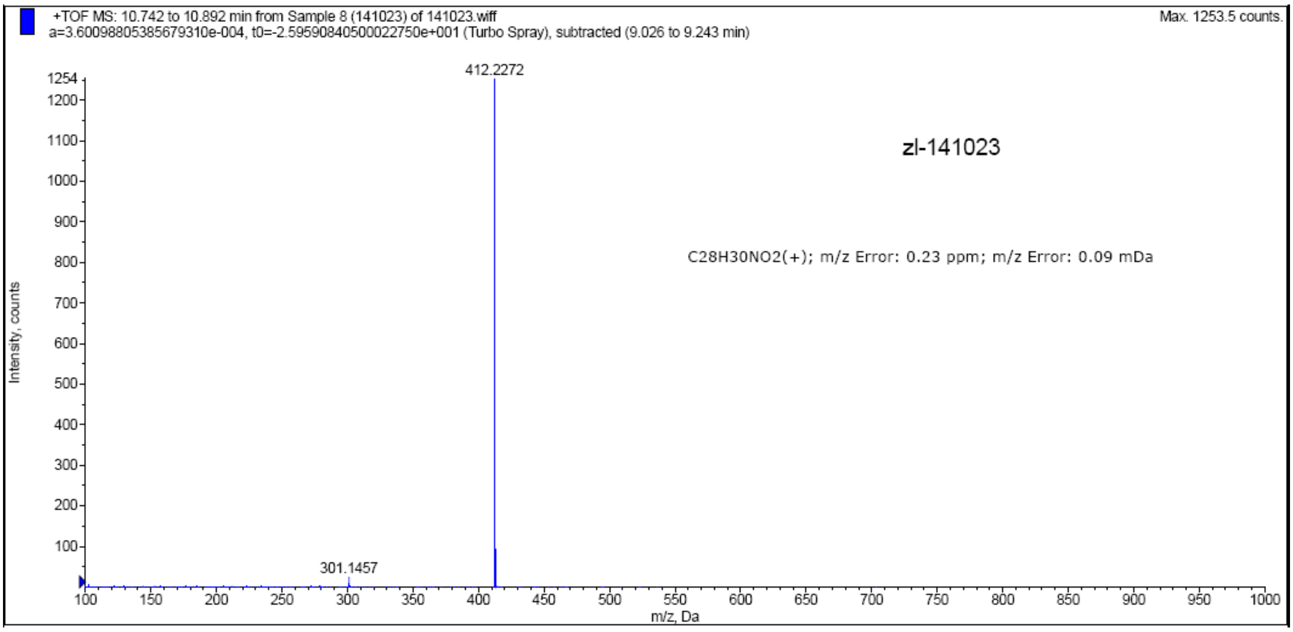

Figure S14 HR-MS identification of compound 1 (calculated for $C_{28}H_{30}NO_2$ $(M)^+$ 412.2271; found 412.2272).

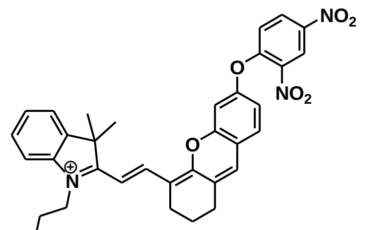
NO2
O
NO2
O
N

NIR-HS

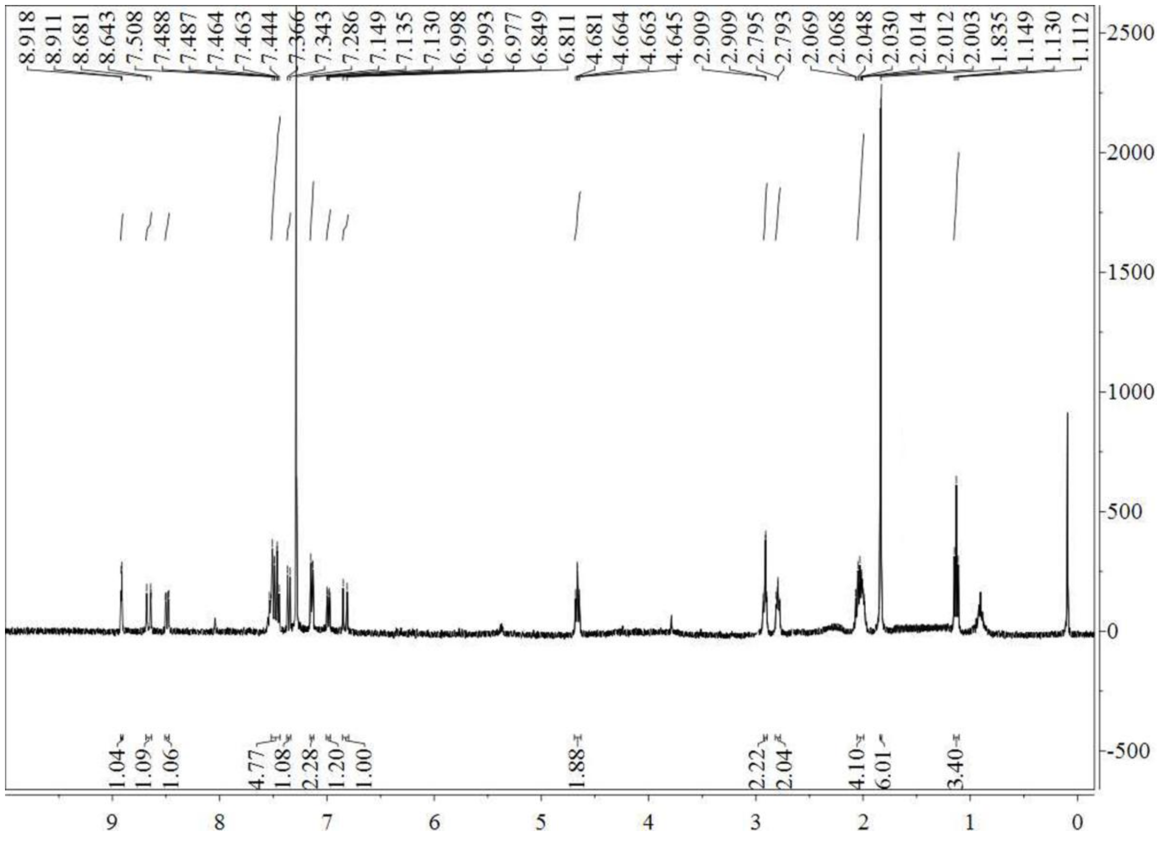
8.918 8.911 8.681 8.643 7.508 7.488 7.487 7.464 7.463 7.444 7.366 7.343 7.286 7.149 7.135 7.130 6.998 6.993 6.977 6.849 6.811 4.681 4.664 4.663 4.645 2.909 2.909 2.795 2.793 2.069 2.068 2.048 2.030 2.014 2.012 2.003 1.835 1.149 1.130 1.112
1.04 1.09 1.06 4.77 1.08 2.28 1.20 1.00 1.88 2.22 2.04 4.10 6.01 3.40
2500 2000 1500 1000 500 0 -500
9 8 7 6 5 4 3 2 1 0

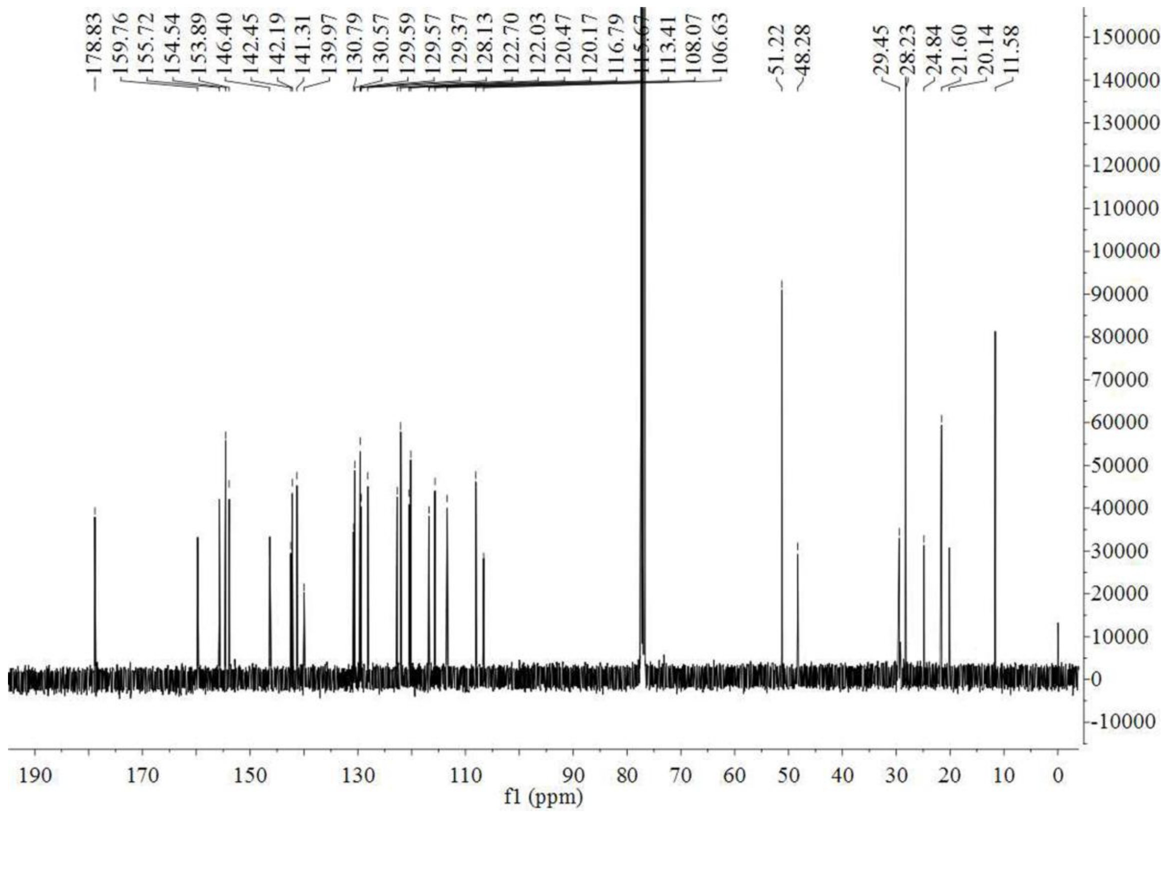
178.83 159.76 155.72 154.54 153.89 146.40 142.45 142.19 141.31 139.97 130.79 130.57 129.59 129.57 129.37 128.13 122.70 122.03 120.47 120.17 116.79 113.41 108.07 106.63 51.22 48.28 29.45 28.23 24.84 21.60 20.14 11.58
150000 140000 130000 120000 110000 100000 90000 80000 70000 60000 50000 40000 30000 20000 10000 0 -10000
190 170 150 130 110 90 80 70 60 50 40 30 20 10 0
f1 (ppm)

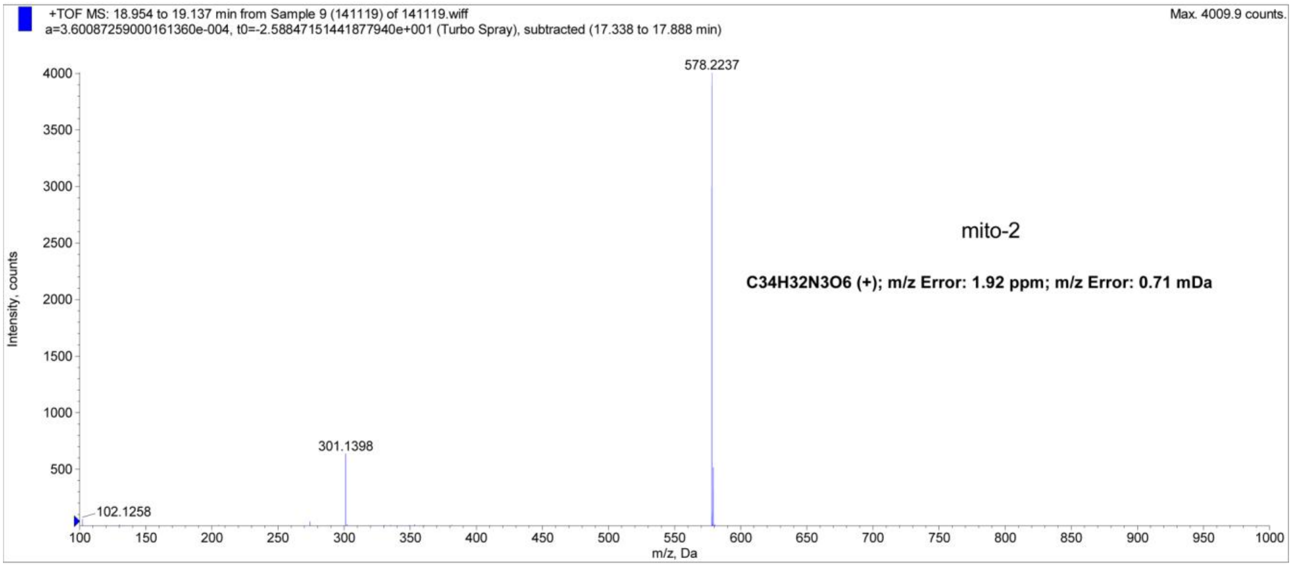

Figure S15 HR-MS identification of NIR-HS (calculated for $C_{34}H_{32}N_3O_6$ $(M)^+$ 578.2286; found 578.2237) .

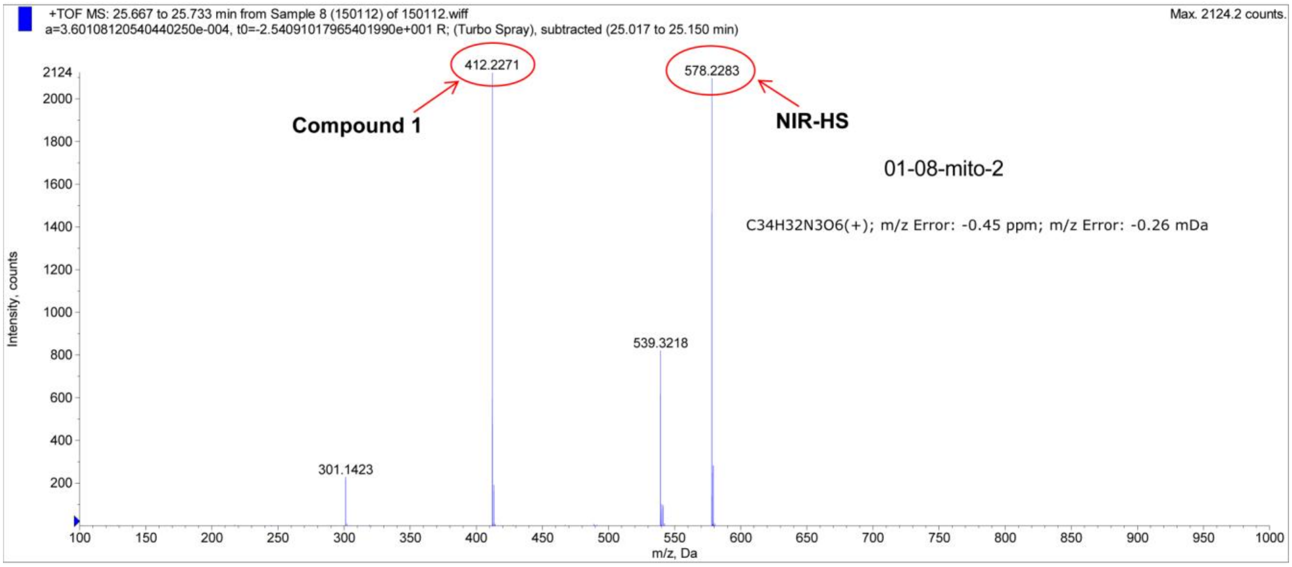

Figure S16 HR-MS identification of NIR-HS + Na_2S.

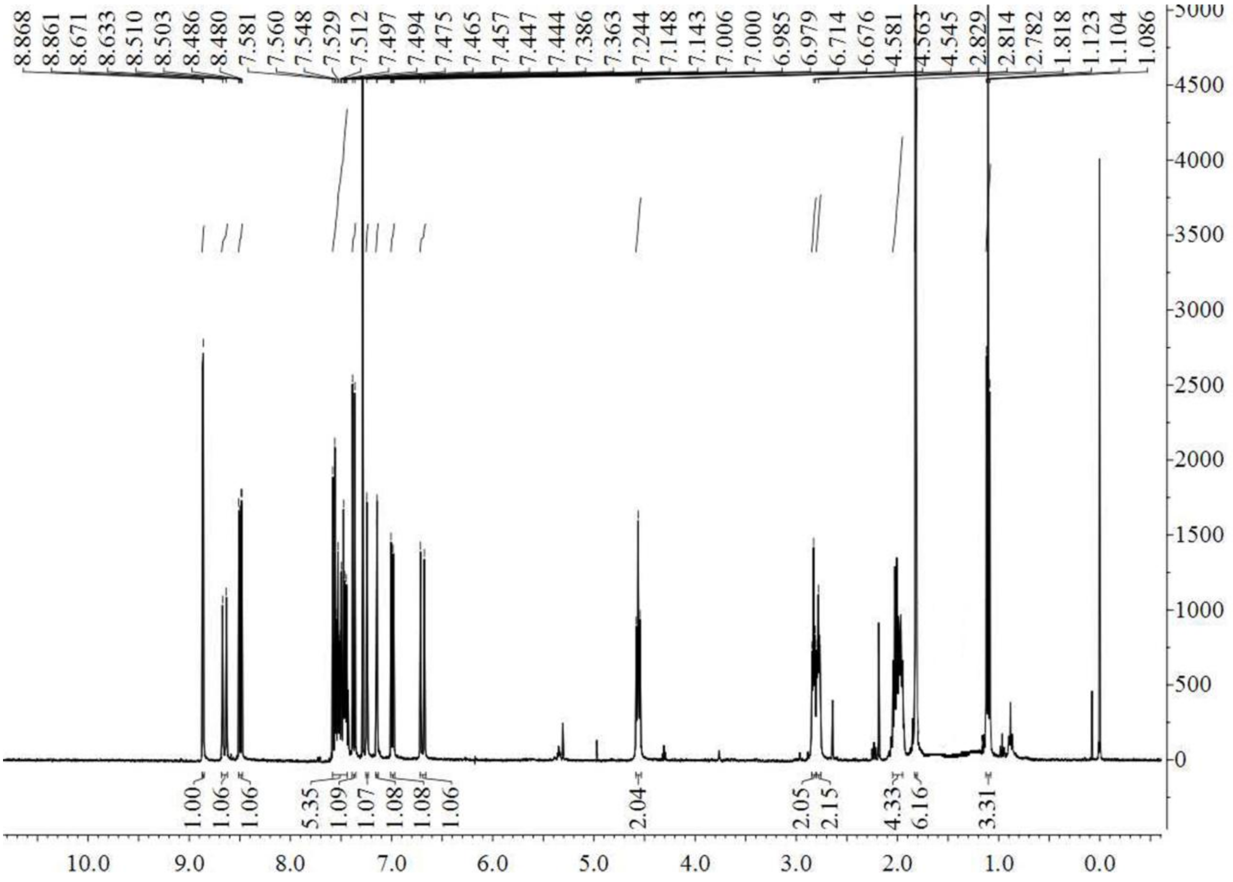

Figure S17 ^{1}H NMR spectra of the isolated fluorescent product of NIR-HS + Na_2S in $CDCl_3$.
